# The Infant Feeding Genogram: a tool for exploring family infant feeding history and identifying support needs

**DOI:** 10.1186/s12884-016-1107-5

**Published:** 2016-10-19

**Authors:** K. L. Darwent, R. J. McInnes, V. Swanson

**Affiliations:** 1 School of Health Sciences, University of Stirling, Stirling, FK9 4LA UK; 2 School of Natural Sciences, University of Stirling, Stirling, FK9 4LA UK

**Keywords:** Infant feeding, Breastfeeding, Family, Significant others, Support, Genogram, Genealogy, Family tree, Tool, Assets based approach

## Abstract

**Background:**

Family culture and beliefs are passed through the generations within families and influence what constitutes appropriate infant care. This includes infant feeding decisions where a family history and support network congruent with women’s infant feeding intentions has been shown to be important to women’s breastfeeding experience. This is reflected in breastfeeding rates where women who were not breastfed themselves are less likely to initiate and continue with breastfeeding. Given the importance of family infant feeding history in the initiation and duration of breastfeeding, and the limited ability of some families to provide support; it is unclear why infant feeding family history and support networks are not explored during pregnancy.

**Methods:**

The Infant Feeding Genogram was adapted from a simple pictorial device that is widely used in psychotherapy and genetic counselling. This tool was developed as part of a study investigating the experience of women when they were the first to breastfeed in their family. Fourteen Scottish participants completed their Infant Feeding Genogram as part of a semi-structured interview. The tool was adapted alongside their narratives to give a visual representation of each participant’s family infant feeding history.

**Results:**

The utility of the genogram is illustrated through two contrasting case examples with very different family feeding histories. The genogram showed family structures, patterns of infant feeding over time, and supportive or conflicting relationships. In the research setting it assisted women to explore their infant feeding history, identify challenges and sources of support and build rapport with the interviewer.

**Conclusions:**

The infant feeding genogram is proposed as a time efficient tool that could assist health professionals and other breastfeeding workers to support women and their families and by stimulating discussion around breastfeeding, Bby identifying strengths or possible deficits in social support for each individual, the tool could inform tailored support and care interventions. The effectiveness and acceptability of the Infant Feeding Genogram requires testing in the clinical environment. However, its successful application in other clinical contexts, combined with the interest in genealogy in popular culture, mean this is likely to be an acceptable, family friendly way to develop more effective breastfeeding conversations.

## Background

### Introduction

The benefits of breastfeeding are well established [1] with health advantages for both baby [2–4] and mother [5, 6]. This underpins the recommendation of exclusive breastfeeding (breast milk only with no other food, milk or drinks) for the first 6 months of life [7]. While these benefits are well recognised and understood by women, the quinquennial infant feeding survey identified that only 24 % of women in the UK (22 % in Scotland) were exclusively breastfeeding at 6 weeks with less than 1 % of women across the UK exclusively breastfeeding at 6 months [8].

There are a number of socio-demographic influences on breastfeeding such as maternal age and education with marital and socio-economic status being highly influential [9, 10]. However, these do not fully account for differences in breastfeeding. A range of social and cultural factors also influence infant feeding choices [11–13], including women’s family and social contexts.

### The significance of families

Most breastfeeding mothers consider social and family support to be important, [14–16] often more so than health service support [17]. A recent study suggested that for some women, family experiences and stories are as valid as, or more valid than, professional advice and research evidence [18]. A congruent social and family network appears to be significant, with support from female relatives, particularly the maternal grandmother, identified as most important and the key source of attitudinal and behavioural norms [17].

The importance of the family context and, in particular, the role of the maternal grandmother’s feeding choice, is reflected in infant feeding statistics. For example, mothers who were themselves breastfed are more likely to intend to, and continue to, breastfeed for longer after the birth compared to mothers who were not breastfed [19, 20]. In addition, non-breastfed women tend to breastfeed for a shorter time [11, 13]. This means that in a context where few women breastfeed, fewer still will breastfeed when they have no breastfeeding family history.

### Potential family influences for successful breastfeeding

There appear to be two main family influences on women’s infant feeding experience; the family scripts that influence breastfeeding intentions and duration, and the ability of the family to support breastfeeding.

#### Family scripts and narratives

Family scripts are patterns of behaviour, underpinned by shared attitudes, beliefs and values which are transmitted through generations and provide guidance about how best to act [21]. Scripts can be ‘replicative’ i.e. doing things the same way that your parents did or ‘corrective’ i.e. doing things differently, often in opposition to the way you were parented [22].

When couples become parents and women become mothers, it appears that they have been ‘bequeathed legacies’ [23] or a, ‘cultural inheritance’, [24] from their own parents, which is then passed on to their own children. These intergenerational legacies influence parenting practice [24] even among parents who do not report feeling close, or in agreement with, their family or who reject their past by choosing another path. To develop their maternal identity at transition to parenthood, women need to make sense of their own upbringing and decide what aspects they value and appreciate and which they will reject [24]. It is also argued that mothers and daughters share an awareness of the embodiment of the process of becoming a mother, a ‘bodily inheritance’ where women assume affinities between their own embodiment and that of their mothers [24–26].

As they approach first-time motherhood, women experience an intergenerational interconnection with their own mothers, enacted in conversations about pregnancy, childbirth and early parenting. This relationship helps to explain the stronger influence of the maternal grandmother over that of her mother-in-law [27]. This may help to make sense of the differences in breastfeeding rates between women who were breastfed themselves and those who were not. For example, where there is limited history of successful breastfeeding in a family, new mothers are likely to be influenced by their mother who may have been encouraged to either bottle feed or breastfed in a regimented manner [28].

#### Family support

Family support is beneficial in terms of increased breastfeeding confidence [29–31] shared breastfeeding experiences [32] and practical suggestions [18]. Conversely, mothers can be undermined by their social network’s lack of knowledge or by negative attitudes and beliefs [33], which may lead to them questioning their ability to breastfeed [34, 35]. This may manifest itself through a lack of emotional or practical support with breastfeeding [36], including receiving conflicting advice from family members who see breastfeeding as an unusual activity which does not easily fit into daily life [37]. Undermining may take the form of either overt, direct criticism or active dissuasion of women from breastfeeding or more covert undermining such as removing themselves from the vicinity of women when they are feeding.

Within the family support network female relatives [31] and particularly the mothers’ own mother, the baby’s maternal grandmother, appear to be most significant [17, 18, 38] although partners within the nuclear family have a role in influencing and supporting breastfeeding. Variations do occur due to cultural and socio-economic factors with grandmothers’ influence stronger in lower socio-economic groups [39, 40] and when living in close proximity [27, 41, 42].

The importance of grandmother support for women is enacted through encouragement and practical advice [20], empathy and approval where breastfeeding success was found to be associated with a high level of approval from women’s own mothers [34]. Grandmothers who had breastfed were found to have significantly more positive attitudes to breastfeeding [43] and transmitted both practical knowledge of how to breastfeed and confidence that breastfeeding is normal [29, 30, 43, 44].

Given the importance of family relationships on infant feeding it is surprising that women’s family histories and family experiences of breastfeeding are not elicited during pregnancy. A record of family assets and potential risks could be used to enable health professionals or other supporters to provide the tailored support that may be needed particularly for women who lack family experience of breastfeeding.

This paper presents the development and application of an infant feeding genogram, a simple pictorial device to map the family history of infant feeding experience, family breastfeeding stories and the potential level of breastfeeding support.

## Methods

### Study context

The Infant Feeding Genogram, was developed as part of a study to explore the experience of women who were the first to breastfeed in a family, how they make sense of their decisions and how this impacts on their family relationships [45]. The study was conducted in the west of Scotland, an area characterized by high levels of socio-economic deprivation that has persistently had one of the lowest breastfeeding initiation and continuation rates in Scotland [46].

### Study design and recruitment

Fourteen participants were purposefully recruited using social media and a range of informal mothers’ groups and networks. Participants who were included had: initiated and sustained breastfeeding for at least 8 weeks within the previous 3 years; were not breastfed themselves by their own mother; did not have a sister or other close female relative who has breastfed, had regular contact with her family of origin; and, to reduce cultural variability, were white Scottish, and spoke English as their first language. Participants were excluded if they were incapable of giving informed consent or were experiencing postnatal mental illness. The study was approved by the University of Stirling School of Health Sciences Ethics Committee in January 2011.

### Data collection

The genogram was used to record relevant demographic and family information to supplement the collection and interpretation of data generated through semi-structured interviews. As such, it provided a pictorial representation of family infant feeding and the nature of the family relationships, and functioned to develop rapport and initiate a conversation about women’s breastfeeding experience in a family context. The stories elicited were analysed using Interpretative Phenomenological Analysis and the genograms assisted in maintaining the idiographic focus, providing context to the women’s infant feeding situation. The themes elicited are reported elsewhere [45, 47].

**Fig. 1:**
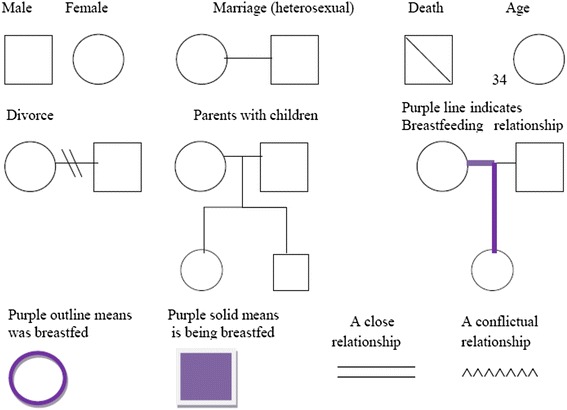
Infant Feeding Genogram Legend

Genograms evolved within systemic family therapy [48]. They are visual representations of information, which show family characteristics, relationships and important life events across generations and have been used extensively as a data gathering and therapeutic tool. Symbols are used to represent family structure, type and nature of relationships, individual characteristics, such as gender and culture, and to record medical information. Genograms record information about families over a three generation period and offer the opportunity to explore family history and their stories over time. They situate the individual in their wider family context, allowing the evaluation of the role of the wider family in difficulties and solutions [48]. Their graphic representation allows complex information to be summarised and easily recognised by practitioners with basic training [49]. There is good evidence for the acceptability of this tool in a range of health contexts [50–52]. It is time efficient as, once familiar with the symbols, it can be completed in 15 to 20 min [53, 54] and it has good inter-interviewer reliability, with different interviewers eliciting almost identical information from participants [55]. For use in an infant feeding context, the genogram was adapted and new symbols developed, as seen in Fig. 1.

#### Completing the genogram

There are four key stages for completing a genogram include: mapping family structure; recording family information such as dates of birth and death, other significant dates, occupations; delineating family relationships including recording strong family bonds or conflicts and finally adding any specialist information such as cultural background or medical history. At each stage, symbols are used to represent the data.

In this study the infant feeding genograms were completed by the researcher (KD). The genograms were completed with all participants at the beginning of the interview and were referred to throughout. It was introduced to participants as a way to organized information about their family, so that the researcher would know who was who during the conversation. The genogram was completed with the participant alone, mapping their family structure, from their perspective, expanding to include others who are not present. This included their current and past marital or cohabiting partnership relations, children from any of these relationships, their current partner’s parents and other relatives from their generation. Then paternal and maternal grandparents and any significant others were added, for example, godparents or close family friends are added. Relevant dates, symbols to represent the nature of relationships, such as close or conflictual relationships, and other information about people who were important to them were then added, as appropriate. In our study the time taken across 14 participants to complete an adapted genogram varied from around 8 to 18 min. All participants completed the genogram enthusiastically and did not express any reservations about the process.

In this study a number of new symbols were developed so the genogram can be used in an infant feeding context. This was done after the completion of the interviews when it became apparent that the existing symbols did not provide a clear enough visual representation of the data. This involved converting notes and black ink into a series of coloured symbols and lines, which could be more easily interpreted. The ease of interpretation was assessed, post study, through consultation with breastfeeding supporters and midwives as part of the research dissemination process, and no changes were required to be made.

A purple outline around the symbol identifies those individuals who have been breastfed and a solid purple shape represents a child who is currently being breastfed. A purple line connects those who have experienced a breastfeeding relationship. This allows for the easy observation and tracking of infant feeding experience within and through families.

It could be argued that as breastfeeding is the biological norm it should be unmarked on the genogram, being the presumed feeding method, while formula feeding should be indicated as the deviation and health risk. However, this would mean that where infant feeding history was unknown the genogram would appear to suggest that breastfeeding had occurred. Given the predominance of formula feeding as the cultural norm, the decision to indicate breastfeeding avoids a potentially misleading situation and ensures the genogram is uncluttered and legible.

The use of symbols to mark breastfeeding does not allow for the representation of breastfeeding duration or the quality of the experience. For example, breastfeeding for 3 weeks and having a very negative experience would be indistinguishable from a year long positive experience. This can however be rectified with additional comments written beside the symbols, or potentially new symbols could be introduced as a development of the genogram. For example, further adaptation of the existing symbols to represent difficulties is possible, for example, a difficult or conflictual relationship is represented as a zigzag line therefore a purple zigzag line for breastfeeding could be used to represent a difficult breastfeeding relationship that was unresolved.

## Results

The Infant Feeding Genogram set the context for a discussion about women’s experience of breastfeeding within their family culture. Through its completion, women spoke of their relationships and family breastfeeding history. While this included factual information, it also elicited detailed recollections of how breastfeeding was for them. In large part, they described a family culture that was unsupportive or actively hostile to breastfeeding. This involved open criticism about their decision to breastfeed and undermining of their confidence and ability to parent. In other families, and sometimes alongside the more open criticism, there was covert criticism of breastfeeding with ordinary parenting difficulties being attributed to the infant feeding decision. Relationally, the criticism often affected women’s ability to seek support from family members and affected how they felt about them.

**Fig. 2:**
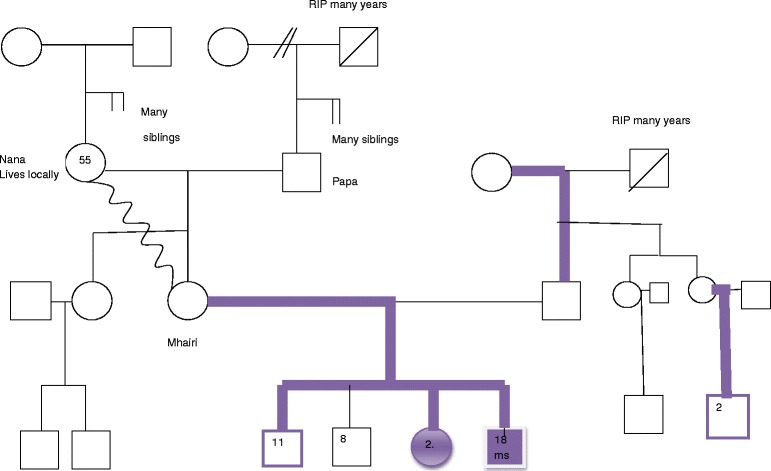
Mhairi’s Genogram

**Fig. 3:**
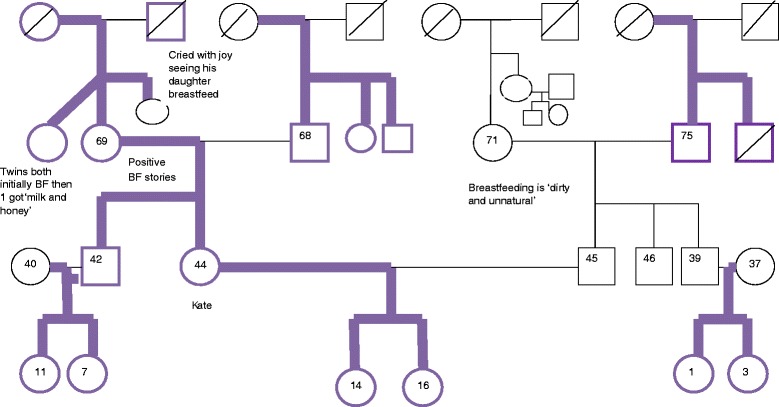
Kate’s Genogram

Two case examples, one from the study and a contrasting example drawn from one of the author’s experience, are used to illustrate the utility of the genogram and the range of breastfeeding characteristics and stories with different families. All names within the genograms are pseudonyms and potentially identifying details have been removed or modified and both individuals gave informed written consent before participating. Mhairi’s infant Feeding Genogram, which was completed as part of our study, is used to illustrate the interpretation of a genogram (Fig. 2). This is contrasted with Kate’s genogram, where there is a strong family history of breastfeeding (Fig. 3). The very different infant feeding histories in these families is likely to impact on their attitudes towards breastfeeding and ability to offer support to breastfeeding family members, and therefore a different, tailored approach is going to be needed in each case.

As can be seen in Mhairi’s genogram, she is married to Gary and has four children aged from 18 months to 11 years old. At the time of interview she was tandem feeding her youngest two children. She has one brother, who is married with one child, who was not breastfed. Mhairi’s parents have a large number of siblings, the number of which Mhairi does not know. Mhairi has many cousins, a number of whom she is in touch with, none of whom have breastfed. Despite positive relationships, they are not supportive of her decision and find the idea of breastfeeding strange, even somewhat repulsive:

‘*I am very close to my cousins, so say we are meeting up for coffee and stuff and she was needing [to be] fed at first they were like, ‘Oh, I don’t know how you can do that’ and, ‘Is that not sore?’*

Mhairi’s maternal grandparents are still alive, as is her paternal grandmother, all of whom live locally. Mhairi’s husband, Gary, was breastfed, as can be seen on the genogram, but Mhairi did not ever discuss infant feeding with her mother-in-law. Gary has two siblings, one of whom breastfed their child shortly after Mhairi had her third child and had shared with her a positive breastfeeding experience. Mhairi sees herself as a breastfeeding role model and this sister-in-law’s breastfeeding decision is seen by Mhairi as a triumph. Mhairi does not know any other details of Gary’s family history. Mhairi describes her family as working class.

Mhairi’s relationship with breastfeeding is complex and she described it as an ‘emotional journey’. She detailed her breastfeeding stories with each child, clearly demonstrating the importance of this participant’s breastfeeding experience with previous children, on her decision making with each new baby. Mhairi had breastfed her first child for 6 weeks and had suffered dreadful pain, ‘agony’, as she described it, with cracked nipples and she blamed herself for not being able to breastfeeding and have a happy baby. After 6 weeks, she stopped with huge feelings of regret and failure. The experience was so awful that she made the decision to formula feed her second child from birth, but suffered feelings of guilt as a result. With her third child, who was born 5 years later, she initially felt very conflicted about her decision and did not plan to breastfeed again as she could not face the disappointment of not succeeding, as she had’ ‘regretted it for years and years’, preferring to make a decision that was in her control. However, after initiating breastfeeding in hospital, she decided that; *‘I’ll maybe give it a go and if it does’nae work out, it does’nae work’, and resolved that she would not, ‘beat myself up about it’.*

Mhairi’s infant feeding experience was one of the most difficult as she faced widespread hostility and opposition to her breastfeeding decision from all members of her family. As she lived close to most of her relatives and was in frequent contact with them, she was bombarded with negative and undermining comments, right from the start of her breastfeeding journey. This includes expressions of revulsion:

*‘Even like just in my mum’s house with family about, because I got the ‘Eugh, can you put that away, its gads [revolting] and stuff, put that baby on a bottle and stuff and it was’nae nice.’*

She found this negative response particularly distressing when the lack of acceptance was from her mother and father. Mhairi details:

*‘I can remember my dad coming into the hospital just after Kevin was born and em, I was feeding him, and I was sitting there all proud and my dad walked in and he was like that ‘Put that away before I come and look at this baby’ like he could’nae come and see the baby, which I understood because they had not see them, anybody breastfeeding or anything.’*

Despite her hurt at this experience, Mhairi tries to make allowances for her father’s harsh response, understanding that this was a new experience for him. While her parents did not ever come to accept or support her decision, their response to it weakened. However, her early days breastfeeding her third child were marred by an incident with her own mother, with whom she already had a difficult relationship, as indicated by the zig-zag line on her genogram:

*‘I had fall outs with people in my family, once I had Chelsea, so I did, I fell out with my Mum because she was needing [to be] fed at my mum’s work when I was out showing the baby off and my mum tried to hide me. She stood in front of me and tried to hide me and told me to hurry up because there was folk coming in, and that was in a council run area, so I know it was breastfeeding friendly, so I fell out with ma mum for oh, we did’nae speak for a couple of months, because it was my choice to feed, she was mine, so it is my choice, but once again, I never had any support, em, because like you expect support off of family, but my mum never done it so she could not understand why I was doing it. It’s weird. It is horrible to think back.*

Even now, many years after this first incident, Mhairi’s distress is palpable and she feels the need to assert her right to have breastfed her own baby, even recounting the location was breastfeeding-friendly, to support her actions. Her disappointment at not receiving the support she felt she needed, when she was really struggling to feed her first baby, remained long after the incident and she felt her parents never really accepted her decision or changed their negative views about breastfeeding, rather they just ‘put up with it’. Despite this negative view, Mhairi would still try to elicit support from her family, but this often resulted in further undermining of her breastfeeding intensions:

*‘She (her mother) could’nae understand it, so I always tried to speak to her about it, so I did and like tell her because she used to say ‘How do you now they are getting enough milk?’ Chelsea never slept for a full hour and I would wake up and my mum would maybe phone and say ‘How is she this morning?’ and I would say she was up ten time and all I do is feed her and my mum would say, ‘Is it no about time you put her on a bottle, maybe she is not getting enough milk?’*

In a clinical context, the information presented in this genogram could be used to determine any specialist support measures, to support Mhairi to meet her breastfeeding ambitions and improve her experience in her family context. This might include additional skilled support to assist her with the physical difficulties she experienced and could get no help with, but also peer support to normalize her experience and build a network of support which is coherent with her intensions.

In contrast, Kate’s Infant Feeding Genogram featured in Fig. 3 has a very different appearance. This shows a strong breastfeeding history in the maternal side of the family, but a more mixed picture in the husband’s family.

Kate lives a short distance from her family and is in regular contact with her mother, visiting several times a month and phoning at least weekly. Kate was breastfed, as was her younger brother, despite the fact that he was premature and the hospital ‘did not recommend it’. She grew up seeing her aunt feed her two younger cousins and two of her older cousins had breastfed their babies before she had her own children. During her pregnancy she reconnected with her own mother through stories of their shared breastfeeding history. For example, hearing about the nostalgic tear Kate’s grandfather shed, seeing Kate being fed by his daughter, who was now a mother herself.

Despite having a difficult breastfeeding experience with her first child, she received emotional and practical support to breastfeed and at no point did she think she would not succeed. Kate’s husband was not breastfed but was supportive of her decision, despite his mother’s very strong views against breastfeeding, which she described as ‘dirty and unnatural’. Despite this negative attitude, the support Kate received from her own family was able to counteract this negative view and she felt able to breastfeed even in her mother-in-law’s presence. Kate’s sisters-in-law both went on to breastfeed their babies.

Kate had very few identified support needs as she had had a history of positive breastfeeding stories and access to emotional and practical support with breastfeeding and early parenting. After feeding her first child, Kate went on to train as a peer supporter, working in a community breastfeeding service, to help other women who had not had the support network she had herself. Rather than needing support herself, she was able to use her own family experience and assets to assist others.

## Discussion

The application of the genogram in the context of infant feeding is novel, however, as a reliable and acceptable practice based tool in a number of health contexts [50–52, 55] its potential in relation to infant feeding support warrants further research. There is growing evidence for family centred approaches to supporting breastfeeding [45, 56–58] and it has been argued that exploring family history of breastfeeding to allow women time to discuss their concerns and fears and/or a group consultation with other family members might be useful [59]. UNICEF UK’s Baby Friendly Initiative also advocates moving away from a ‘tick box’ style of information giving to an interactive and meaningful conversation between midwives, women and their families, which equips them with the skills and confidence to feed their baby [60]. There is, however, little guidance about how this might be done. Our study shows that the genogram offers the potential to create a woman-centred individualized record that can be developed to identify sources of support forwhere women may be at risk due to lack of family experience.

Given the importance of family infant feeding history and attitudes in both the initiation and duration of breastfeeding [20], and the limited ability of families to provide support [36, 37], it is surprising that family history and experience of breastfeeding are not currently recorded or explored in antenatal visits in the UK. This limits the ability of health professionals, or other supporters, to either identify possible assets in the woman’s social network or provide the tailored interventions and support that may be needed by women when feeding their babies.

In Scotland, an assets based approach to health improvement has replaced the older deficits model [61]. This recognises that, in addition to individual assets such as resilience and self-esteem, community assets such as family and friend networks, intergenerational ties and community cohesion are important. It also works from the basis of individual and community strengths to work with people in ways that are person centred and empowering. Its intention is also to reduce reliance on public services and to enable communities to be more self-supporting [61]. Potential challenges to identifying & recording assets (or social capital) and developing person and family-centred breastfeeding support [56, 57], alongside increased pressure on health professionals’ time, indicates the need for efficient and effective tools such as the Infant Feeding Genogram. If used properly this could develop meaningful conversations, build on women’s assets and focus tailored interventions and support to where it is most needed. The growing interest in family history and familiarity with family trees, which share some features with genograms, suggests that this is likely to be an acceptable mother and family friendly way to open up breastfeeding conversations.

While health professionals and other supporters could use these conversations to note family strengths and consider how best families could offer support, there is also the opportunity to ‘troubleshoot’ difficulties. The completion of a genogram could uncover negative attitudes towards breastfeeding and these can be explored in a safe context. By using their listening skills, health professionals could assist families to hear each other’s positions and offer information to challenge breastfeeding myths, possibly allaying difficulties. This might include identifying and discussing family’s stories about early parenthood and expectations of babies’ behaviour, or facilitating a woman to think about how she might ask for assistance in a way that supported, rather than undermined, her breastfeeding intentions.

If relationships are less positive or where the extended family do not want to be involved, the Infant Feeding Genogram can be adapted to these circumstances as it can be used when the family is actually present, or where it is ‘kept in mind’ when working with an individual woman or couple [62]. Its flexibility means that it can be completed with a group of family members, the woman and her partner or the individual woman, making it useful even when the wider family is reluctant to engage.

### Limitations and recommendations for further research

This research was completed in one community in Scotland and recruited women from socio-economically disadvantaged backgrounds where there was no immediate history of breastfeeding; therefore, further testing with more diverse populations is required in order to establish generalisibility. The acceptability and effectiveness of the Infant Feeding Genogram has been established in other contexts; however, the differing skills base between healthcare practitioners and those in the therapeutic context in which it was developed suggest further, assessment is required. Concerns have been raised about the attitudes and knowledge of health professionals, and the impact of their own personal beliefs on their ability to deliver skilled breastfeeding support [63]. This, combined with the recognized gap in the ability to offer emotional support and use active listening [64] may mean that completing a genogram in a meaningful, supportive way may pose some professionals with challenges.

## Conclusion

This paper demonstrates the use of a simple pictorial device, the genogram, in a novel health care context and proposes its use for beginning a meaningful conversation with women about their infant feeding history, stories and culture. The genogram can then be used to explore women’s assets and identify those who may need additional support to breastfeed successfully. The genogram could provide a framework for midwives, and others involved in women’s care to collect information, discuss infant feeding history, stories and culture and begin to challenge these. It would give the opportunity for women, either with their partner or individually, to consider their family support networks and encourage them to begin conversations with their family about breastfeeding and to identify their breastfeeding support needs. Where family support is not forthcoming, this information would allow women to develop early relationships with community peer support services to build a network of social support coherent with their feeding intentions. It would also help health professionals to prioritise services for women who have little support and who may need assistance to succeed in meeting their own breastfeeding ambitions.

Breastfeeding promotion and support has been the subject of several decades of investment and effort yet rates in the UK have shown limited improvement, particularly in terms of duration beyond the first few days. There is an urgent need to develop new and accessible methods of providing women with individualized support according to their needs and particular breastfeeding situation. Our early work using the infant feeding genogram indicates that it has good potential to change the focus of health professional’ interactions to a more dynamic woman-centred approach with further opportunity to identify those most at risk of early cessation.
